# When to Warn? Comparing Heat Indices to Evaluate Public Health Risks

**DOI:** 10.1289/ehp.118-a35b

**Published:** 2010-01

**Authors:** Harvey Black

**Affiliations:** **Harvey Black** of Madison, Wisconsin, has written for *EHP* since 1994. His work has also appeared in *Environmental Science & Technology*, *ChemMatters*, and the *Milwaukee Journal Sentinel*

Summer heat waves can be deadly, particularly for vulnerable populations such as the elderly. Prior to a heat wave’s arrival many cities warn their residents to take precautions such as making sure they drink enough water. But what are the best criteria for issuing a warning of an impending heat wave? A team of scientists from the New York City Department of Health and New York University Medical School compared different metrics used to predict these potentially lethal events **[*EHP*** **118:80–86; Metzger et al.]**. They found that New York City’s current method of basing advisories on the maximum heat index provided a realistic prediction of mortality risk during hot weather.

New York City is one of several places where alerts of excessive heat are triggered by rises in the maximum heat index, a combination of heat and humidity conditions that are forecast for the succeeding 24–48 hr. Alerts typically are issued when the maximum heat index is forecast to exceed 100–105°F (depending on location); some meteorological judgment can be applied by National Weather Service regional staff in whether to issue a heat alert.

In other cities, alerts are triggered by certain spatial synoptic classification (SSC) categories. Under the SSC system, the dominant local weather pattern is categorized into one of several types depending on temperature, dew point, wind direction, wind speed, and cloud cover, as measured four times daily. The SSC categories classified as potentially dangerous weather patterns are determined for a local area by calculating the historical number of deaths in the local region associated with those weather patterns.

The researchers evaluated models using the maximum heat index, the SSC, and maximum, minimum, and average temperatures to predict heat wave deaths in New York City between 1997 and 2006. They found the National Weather Service maximum heat index provided the most reliable prediction of heat-related deaths as confirmed by mortality data from the city’s Office of Vital Statistics, with a spike in the magnitude of the heat–mortality association at maximum heat indices of 95–100°F. Using more variables such as wind speed and precipitation in forecasting heat waves improved the predictive models slightly but also complicated the task of translating complex forecasts into meaningful public health messages.

The authors conclude New York City officials should continue to issue heat alerts when the maximum heat index is forecast to exceed 95–100°F. They also say that repeated warnings should be issued throughout the heat wave and as the maximum heat index increases. Before other cities adopt use of the maximum heat index, however, they should conduct their own analyses with local data.

## Figures and Tables

**Figure:**
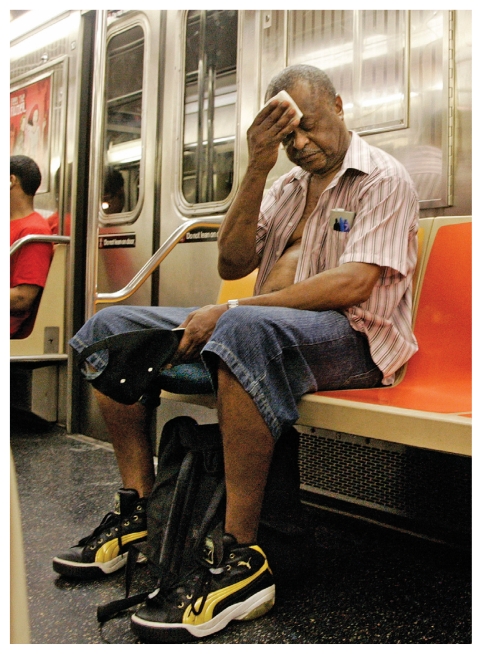
New York City heat wave, 2 August 2006

